# Recurrent tumefactive demyelinating lesions in an elderly woman

**DOI:** 10.1016/j.radcr.2022.09.008

**Published:** 2022-09-28

**Authors:** Erika L. Weil, Mohammad Obadah Nakawah

**Affiliations:** Stanley H. Appel Department of Neurology, Houston Methodist Neurological Institute, 6560 Fannin St, Scurlock Suite 802, Houston, TX 77030 USA

**Keywords:** Tumefactive demyelination, Neuroimaging

## Abstract

Here we describe a 72-year old Caucasian woman who presented with progressive left hemiparesis and hemisensory deficits due to a pathology-confirmed tumefactive demyelinating lesion in the right frontoparietal region. Symptoms improved with glucocorticoids and plasmapheresis, but five months following initial presentation, the patient developed right visual field deficits and acute encephalopathy. Brain imaging revealed near resolution of the initial lesion with interval development of new multifocal tumefactive demyelinating lesions. This case highlights several atypical features associated with tumefactive demyelinating disease, including an older age of onset and recurrent, treatment-resistant lesions. Clinical and neuroimaging features which may be helpful in diagnosing this rare disorder are reviewed.

## Introduction

Tumefactive demyelinating lesions (TDL) are described as large (>2 cm in diameter), tumor-like demyelinating lesions in the central nervous system (CNS). TDL represent a rare subtype of CNS inflammatory demyelinating disorders, though multiple sclerosis (MS) accounts for the majority of cases [1]. Often, TDL present a diagnostic challenge as the radiographic appearance can mimic other space-occupying lesions, including neoplasm or abscess. Patients with TDL may experience a fulminant course with need for aggressive management [2]. While the majority of patients with TDL later follow a typical relapsing-remitting MS course, a small subset exhibit a tendency for recurrent tumefactive demyelinating attacks [1,3]. Due to the overall rarity of this disorder, the immunopathogenesis remains poorly understood. Herein, we describe a case of relapsing TDL which highlights atypical features and an aggressive clinical course.

## Case description

**Fig. 1:**
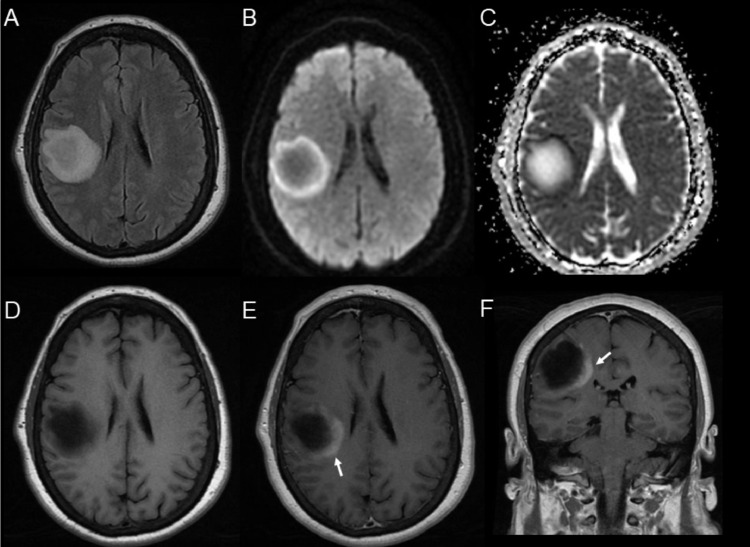
Initial MRI demonstrating a right frontoparietal mass with partial ring-enhancement and mild local mass effect. Axial brain MRI fluid inversion recovery imaging (A), diffusion weighted imaging (B) with apparent diffusion coefficient (C), T1-weighted (D), and gadolinium-enhanced axial (E) and coronal (F) images show a right frontoparietal mass, which measures approximately 4.7 cm in maximum diameter. A mild degree of edema and minimal mass effect are present. A ring of restricted diffusion (B, C) and incomplete ring-enhancement can be seen **(**E, F, arrows**)**.

A 72-year-old right-handed Caucasian woman presented with a 5-week history of progressive left-sided weakness and numbness. Symptoms started approximately 1 week after receiving a second dose of mRNA COVID-19 vaccine (Moderna). Past medical history included obesity, hypertension, hyperlipidemia, nonalcoholic steatohepatitis and erythema nodosum. Neurologic examination revealed mild dysarthria, left facial weakness and moderate left-sided hemiparesis with hemisensory loss. Brain magnetic resonance imaging (MRI) demonstrated a large, right frontoparietal mass with partial ring-enhancement and mild local mass effect (Fig. 1).

Serological studies for infectious causes (including syphilis, HIV, and JC virus) and autoimmune etiologies (including aquaporin-4 and myelin oligodendrocyte glycoprotein “MOG” antibodies) were unremarkable. Spinal fluid analysis revealed normal opening pressure, cell count, cytology, infectious studies, IgG Index, and no oligoclonal bands. Histopathology of the lesion biopsy demonstrated areas of reactive gliosis with dense infiltration of foamy, CD-68 positive macrophages (Luxol fast blue-periodic acid Schiff immunostain positive) predominately involving the white matter with relative preservation of axons identified with neurofilament immunostain. Findings were overall consistent with a non-neoplastic, demyelinating process.

**Fig. 2:**
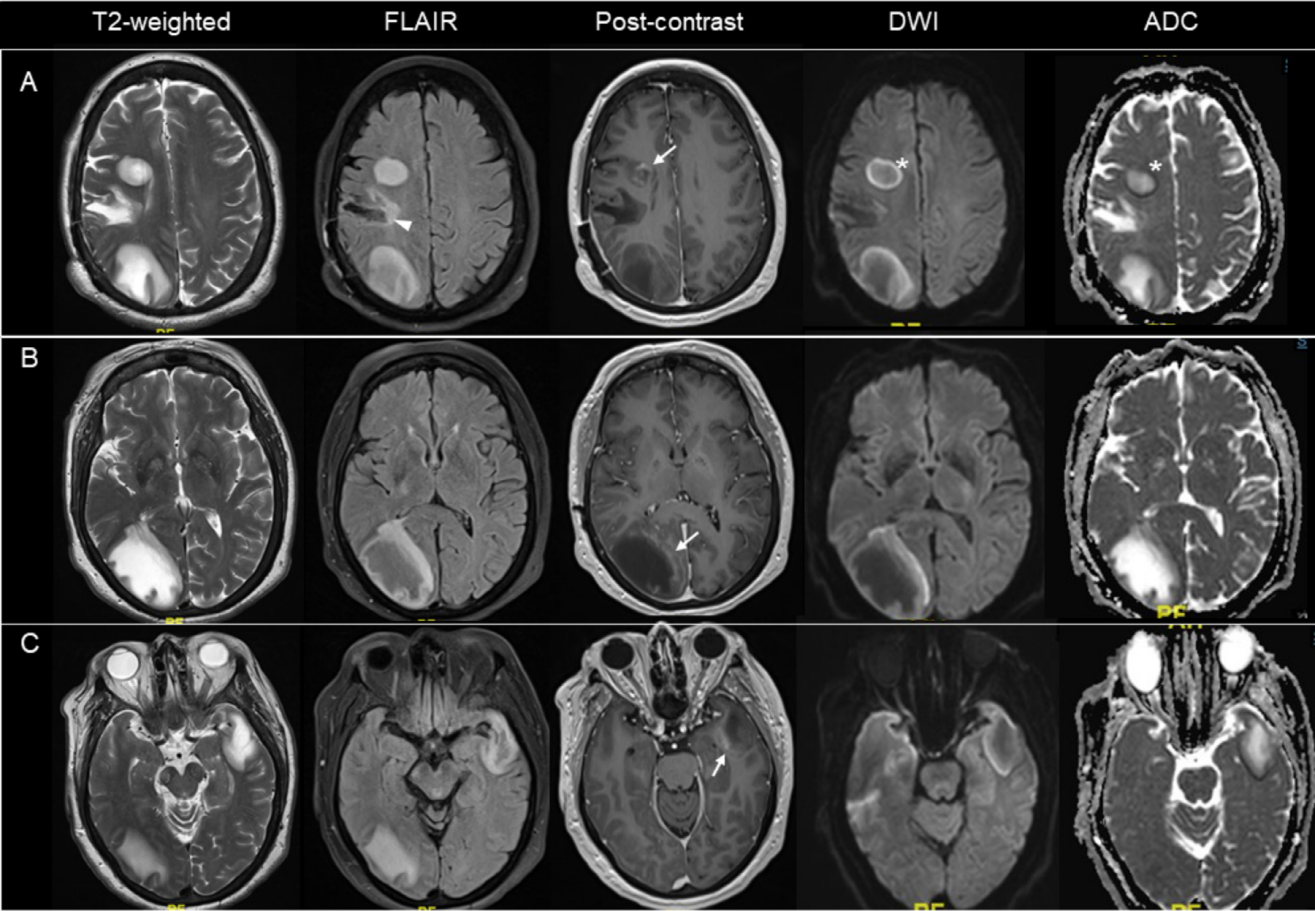
Brain MRI, 5 months following initial hospitalization, demonstrating interval development of multiple tumefactive demyelinating lesions. Axial brain MRI with T2-weighted, fluid inversion recovery imaging, postcontrast, diffusion weighted and apparent diffusion coefficient images (labeled columns) showing interval decrease in size of the previous right frontoparietal lesion (A, arrowhead**)** but development of three hyperintense lesions involving the right frontal centrum semiovale, right occipital lobe and left anterior temporal lobe which have associated edema but exhibit relatively little mass effect. All lesions show partial rim-enhancement on postcontrast images (A–C, arrows**)**. The lesions show peripheral, but not central, restricted diffusion (A, stars).

Following biopsy, a 7-day course of intravenous methylprednisolone was initiated followed by oral prednisone taper. The patient also received 5 sessions of plasmapheresis. She underwent 6 weeks of inpatient rehabilitation resulting in modest improvement of left hemiparesis; estimated Expanded Disability Status Scale (EDSS) was 6. Multiple sclerosis disease-modifying therapy was considered, but unfortunately the patient developed progressive confusion and right visual field deficits prior to starting any disease-modifying therapy, approximately 5 months from initial hospitalization. Repeat MRI showed decreased size of the right frontoparietal lesion with interval development of three new, partial rim-enhancing lesions involving the right frontal centrum semiovale, right occipital lobe and left anterior temporal lobe (Fig. 2). Spinal fluid studies showed mildly elevated white cell count of 21 (74% monocytes, 26% lymphocytes), with normal protein, IgG Index and no identified oligoclonal bands.

**Fig. 3:**
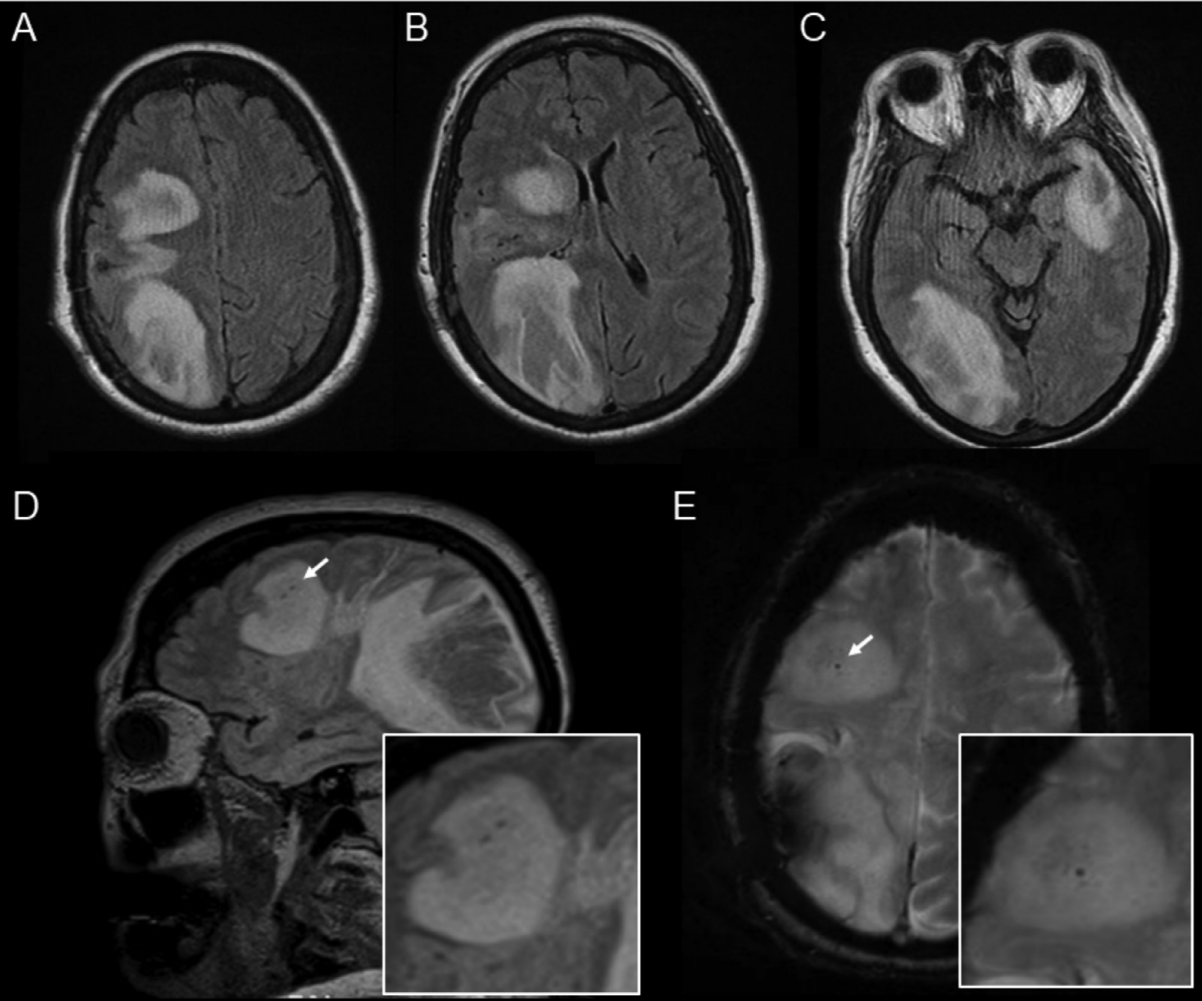
Repeat brain MRI, 11 days following the last imaging, demonstrating expansion of the tumefactive lesions despite aggressive immunotherapy. Axial (A–C) and sagittal (D) T2 fluid inversion recovery images showing interval increase in size of the of the bilateral lesions, now with more prominent edema and regional mass effect. An intralesional central vein sign can be seen on sagittal T2-FLAIR (D, arrow and image inset) and corresponding axial gradient echo imaging (E, arrow and inset).

Despite treatment with high-dose intravenous methylprednisolone and plasmapheresis, the patient continued to decline and ultimately required mechanical ventilation for somnolence and respiratory distress. Continuous EEG monitoring over 4 days demonstrated fewright frontotemporal epileptiform discharges, but no definitive seizures were captured. A repeat MRI brain demonstrated expansion of the multifocal lesions (Fig. 3). Despite a trial of intravenous immunoglobulins followed by rituximab 1000 mg infusion, there was no neurologic improvement. Therefore, per the patient's advanced directive, family elected to forgo further treatments and deferred further life-sustaining measures. The patient was transitioned to comfort care and passed away 2 weeks later.

## Discussion

TDL often present a diagnostic dilemma and may be mistaken for other space-occupying lesions. Patients with TDL commonly have no prior history of demyelinating disease making diagnosis even more challenging [4]. Presentation is typically subacute with multiple symptoms reflective of lesion location and mass effect [1], [2], [3],5]. The estimated prevalence of TDL is 1-3 per 1000 cases of MS and the annual incidence approximately 0.3/100,000 [3,4]. Lucchinetti et al. reported a median age of onset of 37 years in the largest series of biopsy proven TDL; only 4% (n = 7) of patients were older than 65 years at clinical onset [1]. Though uncommon, TDL can occur in older individuals as demonstrated in the present case. When atypical clinical features are present, biopsy of TDL to rule out alternative etiologies may be unavoidable. Despite aggressive management, patients with TDL may experience a fulminant course [1], [2], [3].

The underlying immunopathogenesis of this rare disorder remains unclear. These lesions may be seen in association with a spectrum of other neuroinflammatory disorders such as acute disseminated encephalomyelitis (ADEM), neuromyelitis optica, MOG antibody disease, or tumefactive variant of MS [3,5]. The Marburg subtype is a particularly aggressive MS variant which is often treatment-resistant and leads to a fatal outcome within 1 year [2,5]. It is possible that our patient succumbed to this subtype, though lesions in Marburg variant tend to appear quite destructive and necrotic on histology [5].

Although there is not a pathognomonic radiographic appearance of TDL, certain neuroimaging features may suggest tumefactive demyelination over radiologically similar lesions. TDL are classically 2 to 6 cm in size, and multiple lesions can occur simultaneously [1]. A frontoparietal predilection has been observed [1,6]. Perilesional edema is observed in most cases [1], but relative to lesion size, the edema and mass effect are often much less prominent than what is seen in association with neoplasm or abscess [3]. White matter lesions in a distribution typical for MS can be a helpful clue, if present [3,5]. A hypointense rim on T2 weighted imaging is another supportive radiographic feature [3,^5^,7].

Contrast enhancement is mostly present, though patterns vary. An incomplete ring enhancement with an open rim facing the cortical (pial) or ventricular (ependymal) surfaces may be observed (Figs. 1 and 2, arrows). If present, this is a highly specific sign useful to distinguish TDL from other space-occupying lesions [8,9]. Although nonspecific, closed-ring enhancement is also a commonly observed pattern [1]. Hypoattenuation on a noncontrast CT corresponding to enhancing regions on MRI is another specific sign seen more commonly with TDL than primary central nervous system lymphoma (PCNSL) or primary glioma [9].

Advanced MRI sequences can be of additional value. Blood-sensitive MRI sequences may demonstrate presence of a central vein within an inflammatory demyelinating lesion (Figs. 3 D and E) [7]. Diffusion weighted imaging often demonstrates low apparent diffusion coefficient values along the periphery of TDL with high apparent diffusion coefficient values centrally [3,7], whereas abscesses more commonly show restricted diffusion centrally rather than peripherally.

Recurrence of TDL, as in the present case, is unusual. The majority (70%) of patients with TDL have future demyelinating episodes classic for relapsing-remitting MS after a median of 4.8 years [1]. Similar to this case, a small subset of patients exhibit a tendency for relapsing tumefactive demyelinating episodes [1,3]. No specific determinants for recurrence have been identified [1]. Case reports have described recurrent TDL occurring in patients up to 50-70 years of age [10], [11], [12].

Rarely, immunocompetent patients with PCNSL may present initially with TDL, the so-called ‘sentinel demyelinating lesion’ [3]. Typical neuroimaging features of non-AIDS PCNSL include a solitary homogeneously enhancing parenchymal mass with diffusion restriction due to high cellularity [13], however, this was absent on our patient's imaging (Fig, 2). Additionally, incomplete ring enhancement and other radiographic findings as described above, were more suggestive of TDL.

## Conclusion

This case highlights several clinical complexities associated with tumefactive demyelination. Though uncommon, elderly patients can develop TDL. Helpful neuroimaging clues for the diagnosis of TDL include incomplete ring enhancement and disproportionately mild surrounding vasogenic edema and mass effect relative to the lesion size. Despite aggressive management, a subset of patients with TDL may experience relapses or a fulminant course.

## Ethics approval

All procedures performed in this study were in accordance with the ethical standards of the institutional and/or national research committee and with the 1964 Helsinki Declaration and its later amendments or comparable ethical standards.

## Authors' contributions

All authors contributed to the study conception and design. Data collection, drafting of manuscript and figure preparation were performed by ELW. Interpretation of data and critical revisions of the manuscript were performed by MON. All authors have approved the final manuscript.

## Patient consent

Written informed consent for publication was obtained from the patient's husband.
